# Western Dental Society

**Published:** 1864-07

**Authors:** 


### Proceedings of Societies.

### WESTERN DENTAL SOCIETY.

The Western Dental Society met pursuant to adjournment, at Jacksonville, Illinois, on Tuesday, July 7th, 1863, at 10 o'clock, A. M.

The President being absent, the Society was called to order by the Secretary, when the second Vice-President was called on to take the chair.

Minutes of last annual meeting were read and approved.

The following letter from the President was read by the Secretary:

*Gentlemen of the Western Dental Society:*

No ordinary business could prevent me from being with you at the meeting in Jacksonville, on the 7th inst. I regard it a duty to be there, and regret the circumstance that compels me to be absent. I have taken a deep interest in the success of the Society. This, with other kindred associations, together with dental pericdicals and colleges, has elevated the dental art to the high position it now occupies among the learned professions.

I have had the pleasure to be present at all previous meetings of the Society. It is a privilege I highly esteem to meet my professional brethren, and consult in regard to the best methods of practice. I feel that this is due to our patients, to the profession, and to ourselves. I hope a good number of members will be in attendance.

Gentlemen, of one thing I am certain: socially and professionally you will have a very pleasant interview. I can not close without again expressing my regret that I am not able to be with you.

Most respectfully yours,

*St. Louis, July* 6, 1863. AARON BLAKE.

The Committee on Dentrifice was continued.

Dr. Peebles proposed the name of M. McCoy, of Boonville, Mo., for membership.

Dr. E. Hovey, of Springfield, Mo., was admitted a member.

On motion of Dr. Spalding, dentists present were invited to participate in the discussions.

On motion, the chair appointed Drs. Leslie, Spalding and Hovey an Executive Committee to fill vacancies.

Adjourned to 3 P. M.

AFTERNOON SESSION.

Society met pursuant to adjournment. Minutes of morning session read and approved.

The Executive Committee reported favorably on application of Dr. M. McCoy for membership.

Drs. McCoy, of Boonville, Mo., and Edwards, of Griggsville, Ill., were admitted to membership.

The committee of last year on a certain printed circular and an advertisement put forth by members of this Society, made the following report:

The Committee charged with the consideration of the circular issued and signed by two members of this Society, beg leave to report progress, and to state that while they very much regret that any member of this Society should so far forget what is due both to himself and his fellow-members, as to write and publish matter of the character contained in this circular, still, so far as relates to Dr. H. E. Peebles, they are satisfied that the act was done under peculiar circumstances, and without due reflection on his part. Your Committee being satisfied that no one now regrets the act more than Dr. Peebles himself, are of the opinion that no further action is necessary in his case, and recommend that he be restored to the confidence of the Society.

C. W. SPALDING, }

W. W. ALLPORT, } *Com.*

The treasurer reported $176.94 as the balance on hand. The account was audited by the Executive Committee.

On motion, officers were elected for the ensuing year with the following result:

*President*—H. E. Peebles, of St. Louis.

1*st Vice-President*—H. Barron, St. Louis.

2*d* " " A. Phillips, Missouri.

*Recording Secretary*—C. W. Spalding, St. Louis.

*Corresponding Secretary*—W. W. Allport, Chicago.

*Treasurer*—C. W. Spalding.

*Executive Committee*—W. W. Allport, Chicago, Ill.; C. W. Rivers, Pittsfield, Ills.; S. L. Edwards, Griggsville, Ills.; M. McCoy, Boonville, Mo.; A. M. Leslie, St. Louis.

Drs. Allport and Leslie conducted the President elect to the chair.

The committee on topics for discussion reported progress, and presented three subjects, viz.:

1st. In what cases is it preferable to separate teeth by pressure, and what is the best means for its accomplishment? 2d. When teeth are separated by the removal of a portion of the crown, what instruments are preferable for the purpose, and in what form should the teeth be left? 3d. What instruments and what position of patient and operator are found most convenient and effective for the extraction of the different classes of teeth?

The first subject was taken up and quite fully discussed.

On motion adjourned to 7½ P. M.

EVENING SESSION.

Society met as per adjournment; President Peebles in the chair.

After the discussion of the second topic, reported by the committee, Dr. Allport moved the appointment of delegates to the American Dental Association.

Voted, on motion of Dr. Barron, that one delegate only be sent, and that sixty dollars be appropriated to pay expenses.

On ballot, Dr. Spalding, of St. Louis, was chosen as the delegate.

Art. VII. of the Constitution of the Society was amended by striking out the latter clause.

Voted, that the time and place of holding the next annual meeting be determined by the Executive Committe.

Adjourned to 8 A. M. to-morrow.

MORNING SESSION.

WEDNESDAY, July 8.

Society met pursuant to adjournment. President in the chair. Minutes of last two sessions were read and approved.

The President then addressed the Society as follows:

*Gentlemen of the Western Dental Society:*

I thank you for the honor you have this day conferred upon me, in calling me to preside over you. Ever since the organization of the Society I have felt proud of my membership in it, and have, to the extent of my feeble abilities, promoted its interests, and from year to year I have taken pleasure in elevating to the chair my friends; for in every case I have been successful—for if the man I voted for was not elected, still a *friend* was—for all, I felt, held that relation to me.

But, gentlemen, at no time since the *first* election of officers could you have so honored me as you have done at this time, by testifying to me your preference. I am truly gratified, and while I assure you that I feel my incompetency to discharge the duties of the office in accordance with strict parliamentary rules, nevertheless, with your assistance and forbearance, I will try to do my duty and act impartially.

The good that the Western Dental Society has done to the profession, and for the people in the West, can not be known and fully appreciated by its members. Yet who doubts that the free discussion of modes of practice, and the propriety of interference with natural results in the mouth do tend to good results. The expression oft heard from men of deservedly high position among us, that they have been amply paid for all expense of time, travel and money expended in the attendance of a meeting, by some single idea or thought expressed by a member on the floor, is proof, to my mind, that we are all improved and benefited; and, as a necessary consequence, the people are also greatly benefited.

It is, however, deeply to be deplored that so many worthy men in the profession take little or no interest in any enterprise of this kind, and manifest no desire to impart or receive any new light, but, terrapin like, retire within themselves and close their shells.

It is much more cheering to us, and we think beneficial to dentists, who do not feel inclined to enroll their names on our books, to come into our halls and accept our proffered courtesies, and encourage us by their participancy in our debates; they may not go away unrewarded.

That this Society is destined to live and accomplish much good in years yet unnumbered, who can doubt? for it is now living and even growing through this time of darkness and storm, of war and ruin. True, it is not thrusting out many strong shoots and branches, or putting forth much foliage or bloom, and yet its roots are striking deeper and deeper, and even while the storm rages, it bears some fruit.

Gentlemen, in 1836, when I entered the profession, there was not a single dental college or society of dentists in the WORLD, so far as I knew. It was not an easy matter then to obtain practical knowledge, as every operator had a set of facts for which he paid a high price and which he held very dear, and if he had evolved any new facts or idea wholly his own, he held them still more dearly, and guarded them more jealously. But such is not the case now, in 1863, and no man rejoices more than I do at the change. Dental knowledge is now cheap and free to all who desire to obtain it. Our colleges, our literature, and our associations afford great facilities of acquisition.

Pardon me, gentlemen, for trespassing on your time so long, and again accept my thanks and good wishes.

The consideration of the third topic for discussion was commenced and continued till adjournment.

Adjourned to 3 P. M.

AFTERNOON SESSION.

The Society was called to order by the President, at 3 P. M. Minutes of last meeting were read and approved.

Dr. G. S. Miles, of Edwardsville, was proposed and subsequently elected a member of the Society.

A fourth topic reported by the committee, viz.: "The general subject of filling teeth," was then taken up, and discussed till near the hour for adjournment.

On motion the thanks of the Society were tendered to the proprietor of the Dunlap House for the use of a large and convenient room for the meetings of the Society.

On motion adjourned, to meet at time and place to be designated hereafter by the Executive Committee.

C. W. SPALDING, *Recording Secretary.*

---

### *Discussions.*

### FILLING TEETH.

Dr. C. W. Spalding, of St. Louis, opened the discussion on this topic as follows:

The subject of *filling* cavities, including the various modes of manipulating foil, &c., &c., has been very fully considered of late years by the different Dental Associations in this country. But so far as has come to my knowledge, that important part of the operation which relates to the proper preparation of cavities for receiving the gold, or other material used in filling, has not received that attention which its importance seems to demand. We now propose to consider this particular division of the operation of filling teeth, believing it to be one upon which the ultimate success and durability of the succeeding steps in the work in a great degree depends.

One of the most important objects in the preparation of a cavity is to secure a proper form—one that will retain the filling, and at the same time admit the packing of the material solidly and well into all its parts. The walls should be generally perpendicular to, or at right angles with either the face of the plug or the floor of the cavity. In order to obtain this form, it is sometimes necessary, when the cavities occur in the grinding surface, to first fill such undercuts as are protected by a strong overhanging shelf of enamel. And just here allow me to digress so far as to say that having formerly found much difficulty in filling such undercuts with gold, either in the shape of foil or crystal, I have latterly substituted os-artificial for the purpose, and have found it to answer admirably. This substance is easily moulded, and is readily trimmed out into the right shape after it has become sufficiently hardened. Undercuts occurring in the sides of the cavities nearest the gums should be resolutely cut away. They are often met with along the necks of the molars and bicuspids and in all such localities the right angular wall is absolutely required; without it there is no certainty that the work will be durable, however well it may be done in other respects. But in the incisors a groove or hollow may be retained, provided a strong shoulder has been left by the separating file, and a little undercutting is also allowable at the opposite end of the cavity. In very shallow and broad cavities, especially when they are located in the labial surfaces of the incisors, some undercutting is almost necessary, but wherever it is practical the beveling or rounding off of the marginal corner of the cavity is particularly necessary, and should never be omitted. Indeed the removal of this sharp corner around the edge of a cavity is always advisable, unless the filling to be introduced is of some plastic material; then, I should say, the sharper the corner the better.

The instruments required by the operator for the preparation of cavities is very much a matter of taste or of habit. Some operators use, and think they must have, a great variety of patterns and sizes. I use but few. My main reliance is on the hatchet-shaped excavator of different sizes and bent at different angles. One or two other forms are sometimes convenient, not to say necessary. Drills of several kinds, including the burr drills in variety, are very useful, though not indispensable. Every operator must decide for himself what instruments he will employ, discarding only such as do not facilitate the proper shaping of the cavity. Of late years I have made much use of small curved files, both in shaping and smoothing the walls of cavities in the grinding surfaces, and have found them especially useful in rounding out the sharp angles in those having an irregular form.

Dr. J. H. Hyde, of Lewiston, Illinois, suggests that in separating between the lower back teeth, especially the bicuspids, the spaces should widen towards the lingual side, for the purpose of allowing the easy removal of food, and thinks food would generally be dislodged in the act of mastication. In the upper jaw the spaces should widen in the opposite direction.

Dr. Henry Barron, of Saint Louis, prefers a V shaped separation between the back teeth as a general thing; finds more difficulty in the preparation of large cavities in the lateral incisors than in other teeth, especially when the front wall is much broken away; makes narrow undercuts in such cavities; separates freely with file or wedge, or both; prefers cotton for wedging the teeth apart. Thinks a broken excavator makes an excellent drill, the fracture making a good cutting edge.

Dr. I. Forbes, of St. Louis, in the preparation of cavities first cleans the outside of the tooth, so as to determine the extent of inside decay, then cuts away the enamel until he gets a straight line; likes to have the bottom of the cavity as near level as practicable; if fissures occur opens them with a drill; breaks away the enamel as long as it breaks easily, particularly in the side of the cavity towards the gum; often even goes beyond the termination of the enamel for the sake of securing a right angle in the wall of this portion of the cavity. For instruments he uses excavators of three shapes, hatchet, hoe and scoop, together with several varieties of drills, including the conical and inverted truncated conical; employs a burr drill larger than the diameter of the cavity, for the purpose of counter-sinking, previous to filling; has latterly noticed a new description of files which he likes; they are cross-cut, with one cut deeper than the other. In preparing the cavity described by Dr. Barron, he would cut away the front till the enamel was strong enough to fill against; thinks one is liable in these cavities to leave a little diseased bone, and that teeth often re-decay from this cause; never begins to fill any cavity till he can *see* every part of it.

Dr. J. B. Morrison, of St. Louis, employs chisels of various sizes in opening cavities; thinks it important that cavities are counter-sunk to prevent crumbling; takes off just enough to produce a dull edge; does not like undercuts, and avoids them as much as possible; wants the shape such that all parts of the cavity can be easily reached.

Dr. C. W. Rivers, of Pittsfield, Ill., aims at the right angle, or something near it in the general preparation of cavities, but does not object to a little undercutting; has filled undercuts with gold, but likes the suggestion of Dr. Spalding, of filling them with os-artificial; always separates freely, so as to have plenty of room to work in.

Dr. J. G. Nichols, of St. Louis, likes to have the inner diameter of the cavity nearly equal to that of the orifice; takes away all the weak enamel. In the case of bicuspids he uses a moderately thick separating file, and after securing a right angle at the upper side slightly undercuts the lateral walls of the cavity, making it a dove-tail shape, and restores the original form of the tooth as far as practicable; has never employed os-artificial for filling undercuts, but has used it to strengthen walls that were light and thin; uses chisels in separating, and then rounds the corners with a file; thinks Dr. Barron's case one of the easiest of cavities; does not like to see the form of the tooth marred by much filling or cutting away, and hence aims at restoring the shape with gold as far as it is possible to do it; has not used wedges of any kind for several years; even in the case of a child would not wedge at all, but would prefer to cut away as little as possible, and fill with less room to work in.

Dr. Spalding relates a case where death of the pulp of a central incisor followed wedging in the mouth of a lady over forty years of age.

Dr. Forbes questions whether the wedging caused the death of the pulp, and does not think inflammation is produced when wedging is done with care.

Dr. Comstock, of St. Louis, uses no unusual forms of instruments and is not particular about right angles, but must get the plugger in contact with all parts of the cavity; also is not particular about removing all decayed bone from the bottom of the cavity.

VULCANITE BASE.

Dr. Comstock likes vulcanite, especially for temporary work; objects to its thickness, but believes a better adaptation to the gum can be had with this material than with gold; finds that it will not bear re-vulcanizing more than two or three times, and hence can not be many times repaired.

Dr. Nichols esteemed vulcanite as superior to any other material for temporary sets and for partial lower cases; for the latter he leaves the rubber quite flexible. When used for partial upper sets he always inserts an air chamber, but not in full sets. This material is also valuable when the shape of the face needs to be restored; prefers plaster of Paris for the trial plate, instead of gutta percha or wax, as commonly used; fills joints between teeth with os-artificial, and imbeds a strong wire in the plaster, enclosing the teeth to prevent the moving of the teeth when under pressure. He also has a seat valve inserted in the vulcanizer. During the process of vulcanizing, this valve is open and the steam passing through it acts on a test piece of rubber. By closing the valve this test piece can be removed from the boiler and the progress of the vulcanizing process be at any time accurately determined.

Dr. Spalding leaves partial lower sets rather flexible; inserts a strip of gold plate across from side to side, allowing it to extend as far as the first tooth in each block; perforates the plate and covers both sides with vulcanite. Lower sets thus made are not liable to be broken.

Dr. Barron considers vulcanite a useful material as an adjunct; thinks it fit for full sets only, and believes there are cases where vulcanite, owing to its adaptability, is preferable to any other substance; has found much difficulty in repairing, and would rather make a new piece than to attempt repairing an old one.

Dr. Morrison has used vulcanite about three years, and is satisfied that for certain cases it has no equal, and prefers it to any thing else for a majority of full upper sets; finds it too light for lower sets when the case presents a low ridge. If the ridge is high he would use it; does not like it for partial upper sets.

Dr. Rivers was slow to adopt the use of vulcanite; has now used it two years and finds it as good as any thing for all full sets, and likes it very well for partial sets. In the latter class of cases he uses common plate teeth and backs them; thinks the additional thickness over ordinary plates no objection, when the wearer once becomes accustomed to it.

Dr. S. L. Edwards, of Griggsville, Ills., has used vulcanite only one year; employs it for all temporary work, both full and partial sets; finds it can be successfully worn when left quite soft.

Dr. Hyde has always entertained fears of poisonous effects resulting from the substance used in coloring vulcanite, and has not used it at all on that account.

Dr. Cornelius, of St. Louis, does not think vulcanite worthy of general introduction. Coralite is better, for some cases, it can be repaired three or four times, and still be good; sometimes unites the gums of teeth with continuous gum enamel to prevent showing the rubber.

Dr. A. M. Leslie, of St. Louis, says Barker's Solution has been found to color the rubber. A weak solution of oxalic acid in water or alcohol will bleach out the color to a bright red in a few hours; has seen a case worn by a dentist who stated that the rubber was wearing away in his mouth; found it covered with a soft coating which could be removed with the finger nail.

EXTRACTION OF TEETH.

Dr. H. Barron declared himself an advocate of the free use of the gum lancet, preparatory to extraction; thinks he draws an indication from the cutting of the gum of the difficult or easy removal of the tooth itself. These remarks do not apply to deciduous teeth. He has two or three pairs of forceps with which he extracts a large majority of teeth. One is a narrow-beaked instrument, originally designed for removing crowded teeth; another is a pointed beaked lower molar forceps, and the third an upper molar of Maynard's pattern. The only anæsthetic he uses is bourbon whisky. When extracting upper teeth, he stands to the right of the patient, and a little behind when removing the lower. The elevation obtained by standing on a stool lessens the liability of striking the upper teeth; likes to rotate a front tooth when practicable.

Dr. Edwards, of Griggsville, Ill., stands in the same position indicated by Dr. Barron, at the same time supporting the head with the left hand; rarely finds it necessary to use a gum lancet.

Dr. C. W. Rivers prefers forceps of Harris' patterns; uses gum lancet sometimes to gratify the patient's wish; employs the hook in the extraction of roots more than any other instrument; frequently starts the lower dens sapienta with a common elevator, removing afterwards with bicuspid forceps.

Dr. McCoy, of Boonville, Mo., also prefers Harris' patterns; gives brandy in preference to chloroform; uses the lancet freely for the purpose of making free passage for the forceps, and also as a prospecting instrument; rotates front teeth; uses hook mostly for roots, occasionally the punch and screw; regards the latter as indispensable.

Dr. Sawyer, of Jacksonville, Ill., uses forceps of Chevalier's patterns, excepting upper molars, which were made by Rees; employs screw for upper incisors. When making an adjoining tooth serve as a fulcrum, he often gets a block of wood to protect the tooth from injury. Relates a case when he failed to extract a front tooth, and three weeks afterwards, on renewing the attempt, it came out easily. The handle of the punch he uses is long enough to rest against the body when applying the force; thinks it gives better control over the motions of the instrument; has a twice bent elevator, which can be applied from the inside.

Dr. G. S. Miles, of Edwardsville, Ill., has but one forceps for the first ten upper teeth; thinks root forceps give less pain than hooks, punches, &c.; discards the screw and though he retains the use of the gum lancet he does not think its employment promotes the extraction of the tooth.

Dr. Hovey, of Springfield, Mo., is in the habit of separating the gum from the neck of the tooth, by passing a blunt edged instrument down to the alveolar border, and thus making free way for the forceps.

Dr. Black, of Jacksonville, uses a straight incisor forceps for the first ten upper teeth, and a curved one for the first ten lower teeth; rarely uses any thing but forceps, and has extracted twenty-eight teeth from the same mouth without changing the forceps.

Dr. Peebles requires but one forceps for the first ten upper teeth. One forceps, also, serves for the upper molars on both sides. The outer beak of this forceps is pointed; uses the pointed beak forceps for lower molars on left side, and a small hawks-bill forceps for the same teeth on the right side; frequently separates the roots of teeth with a cutting forceps.

Dr. Spalding can only suggest, in addition to what has already been said, the use of the *compound* elevator in the removal of the lower dens sapientia; has always found it a reliable instrument for that purpose.

TREATMENT OF EXPOSED PULP.

Dr. Spalding has not much confidence in any attempt to preserve the life of the dental pulp after it becomes actually denuded. If there remains ever so thin a covering of dentine or cementum, he would certainly advise an attempt at preservation. This is best accomplished by covering the floor of the cavity with some substance which we call a non-conductor, and then completing the operation by filling in the usual manner. But when the exposure of the pulp is complete, he never hesitates to apply at once the means for securing its rapid destruction. When once certain that the pulp is entirely extirpated, including the root canals to their extremities, he proceeds without delay to fill those canals in the most complete and perfect manner practicable. This accomplished, he feels that the tooth is safe, and the subsequent steps in the operation present no unusual difficulties; thinks the want of success often arises from delaying the filling of the root canals; treats a great many cases of this character; has more or less of them in hand constantly.

Dr. Barron fills the roots of teeth in but few cases; prefers the mechanical removal of the pulp without resort to escharotics of any kind.

Dr. Nichols always destroys exposed pulps and also many that are *nearly* exposed. If the overledging matter is not sufficiently thick to admit the use of os-artificial he would apply an escharotic and destroy the pulp at once; is careful to remove the entire pulp, and then fills the pulp chamber in the usual way; does not fill the roots in any case; is generally successful when he feels certain that the root-canals are entirely freed of soft tissue, and can be wiped out with creasote, using it quite freely. When inflammation of the peri-dental membrane supervenes, he makes no attempt to save the tooth, but considers it past remedy.

Dr. Rivers caps exposed pulps successfully with whalebone when in a healthy condition, provided the patient has a good constitution; in other cases he destroys and removes the pulp in the usual manner, and fills the roots to the extremity; considers such teeth, if well filled, of more value than artificial ones. In reply to a question by Dr. Nichols, he answered that he filled the roots for the purpose of closing the foramen at the apex of the root, with a view to prevent the ingress of fluids.

Dr. Forbes remarked that he found it more difficult to succeed with lower teeth than with those in the opposite jaw, owing, as he believed, to the more ready discharge of fluids from the canals in the upper teeth, during the treatment preparatory to filling. When he did not succeed in thoroughly clearing the canals, he dressed with astringents to convert the soft matter into leather. If successful in this he considered the chances good for preserving the tooth.

Dr. Hyde fills canal and pulp chamber with os-artificial, and described an interesting case of diseased antrum.

[*To be continued.*]

